# Supplementary material for: Do bank voles (Myodes glareolus) trapped in live and lethal traps show differences in tick burden?
Source: PLoS One. 2020 Sep 17;15(9):e0239029. doi: 10.1371/journal.pone.0239029 (PMC7498064; doi:10.1371/journal.pone.0239029)
Supplement: Attachment — Submitted filename: Response.docx [file pone.0239029.s001.docx]

**Manuscript PONE-D-20-16817**

**Response to Reviewers**

Dear Dr. Stevenson,

We would like to thank you for the opportunity to submit a revised draft of the manuscript "Do bank voles (*Myodes glareolus*) trapped in live and lethal traps show differences in tick burden size?" for publication in the journal *PLOS ONE*. We are deeply appreciative of the effort and time accorded by yourself and the reviewers providing valuable feedback on our initial manuscript and we greatly welcome all the insightful comments and suggestions on how to improve our paper. We incorporated most of the suggestions made by the reviewers. Those changes are highlighted in the "Revised Manuscript with Track Changes". Below you can find a point-by point response to each of the reviewers' comments and concerns (in blue). All line numbers refer to the new manuscript file with tracked changes (All Markup).

**Reviewers' Comments to the Authors:**

**Reviewer #1**

MAJOR CORRECTIONS

1. Convince us about Why you are interested in ticks on voles in the first place. What pathogens would they be suggested to play a role in transmitting? If something such as Borrelia burgdorferi., then larvae are not likely to be infected due to lack of transovarial transmission. Discuss. What evidence is there of voles being a reservoir for tick-borne pathogens?

**Author response:** Thank you for the suggestion. The second paragraph of the introduction focuses solely on epidemiology and how both ticks and rodents can transmit diseases to eachother, and how in turn ticks can then transmit these to humans, and why this is important. To emphasize this we have added several references on each of the aforementioned statements on lines 66-71. Additionally, a sentence emphasizing the danger of tick-borne pathogens to human health has been added on line 68-69.

2. Why were voles euthanized following capture and tick assessment? Explain why ticks couldn't be collected and then the animal released as a viable option. Improve the introductory rationale.

**Author response:** Thank you for this question and this remark. Ticks could not be collected from live animals as the risk of injury and capture myopathy to the animal is too high. As explained in the methods, a full body analysis of a dead vole took minimum 20 minutes. This was when a vole carried no ticks. As ticks are cemented into the skin, it would be very difficult to remove ticks from a live animal. Some voles carried up to 100 larvae, which meant a processing time of approximately 1h30 min. This would simply be unfeasible on a live animal in a field setting. As mentioned in the introduction (line 58-59), capture myopathy often has fatal consequences, meaning a stressful death.

Another option would be to keep the animal alive and captive, and waiting for all larvae to reach repletion. Considering the large numbers of animals involved in this study, the time and capacity required to achieve this would be far too great.

However, your concern is certainly valid, as efforts should be taken to reduce animal suffering where possible, and avoid unnecessary death. To address your concern, we have added additional motivation in the Methods chapter under "Host trapping" (line 143-148), explaining in more detail why we opted for a full post-mortem examination, rather than capture and release.

Additionally, comparing burdens from lethally trapped voles and live captured voles without a full body post-mortem examination would mean a big difference in sensitivity between observation methods.

3. How was stress in the live-captured animals measured? How do you know they were experiencing stress being held for a period of time.

**Author response:** This is a good question. Field measurements of stress (e.g. blood sampling for cortisol measurements) were not performed. The stress is therefore not measured but assumed from ample literature on capture stress experienced by wild animals.

4. What were the species other than bank voles encountered during trapping?

**Author response:** Thank you for this question. A summary of all encountered species should indeed have been included in the initial manuscript. In order to correct this, we have mentioned all the species we trapped in the first paragraph of the result section (line 213-217).

5. If as found, only larvae would be expected on voles, explain when peaks of Ixodes ricinus larvae generally occur.

**Author response:** Clarification of when larval *I. ricinus* peaks are expected was added on line 391-392: “…whereas larvae generally show a single activity peak in summer to autumn.”

6. To avoid differences in behavior or tick prevalence of populations in the areas sampled, I would have used a mixed grid design, rather than putting lethal and live traps in separate areas.

**Author response:** Thank you for the suggestion. Indeed, a mixed grid design would have been logistically possible. However, during the field work, many different people participated, and only a select few were capable of properly euthanizing animals. In order to ensure that euthanasia happened quickly and swiftly, during the field work the collection teams were split up. One qualified participant would go through the live traps and perform the euthanasia, another would go through the lethal traps and collect lethally trapped animals.

7. Weblink to data sharing wasn't functioning; but maybe this will be activated upon publication.

**Author response:** Reviewer #1 is correct. An embargo is currently in effect on the dataset. This will be lifted upon publication of the paper, and the data will become fully accessible.

MINOR CORRECTIONS

8. TITLE: The word 'size' isn't needed, and to state burden is sufficient. This applies throughout the manuscript.

**Author response:** We appreciate this remark, and we agree. The word 'size' has been removed from 'burden size' in both the title and throughout the manuscript in all but one case (line 91) for the purposes of clarification.

9. Short Title: Suggest 'Comparative tick burdens on voles by trap type'

**Author response:** Will be implemented upon resubmission.

10. I would even consider renaming the main title as "Influences on Ixodes ricinus larval tick burden on bank voles (Myodes glareolus) captured via lethal and live traps".

**Author response:** Thank you for this suggestion for title revision. We have considered this and other options thoroughly, and unfortunately we feel your suggestion is quite long and detailed. We therefore opted to keep the remaining title as we feel it is more to the point, although we removed the word “size” as your proposed under point 8.

11. ABSTRACT: check where commas are needed

**Author response:** Thank you for the observation. We have gone through the remark and two commas have been added on line 38 and on line 41.

12. Check the spelling of 'ricinus'

**Author response:** Indeed, there was a spelling mistake on line 31. This has been corrected.

13. (line 14) - add the word 'tick' before burden.

**Author response:** the word 'tick' has been added to 'tick burden' on line 37.

14. Since you only analyzed larval ticks, please state that within the abstract/title.

**Author response:** Indeed we have only included larvae in this study. 'Larval' has been added to 'larval burden' on line 31 and 'larvae' has been added to '...5.7% more larvae compared to...' on line 37.

15. METHODS: Line 123 - Replace 'for this study' with 'in the analysis'.

**Author response:** 'for this study' has been replaced with 'in the analysis' on line 152.

16. RESULTS: Your key hypothesis/question concerns the trap type effect, so I would move those results up; then later talk about your models of tick abundance factors.

**Author response:** Thank you for the suggestion. The effect of trap type on larval tick burden is indeed the core of the study. We have implemented your suggestion by moving the paragraph on trap effect up in the Results chapter to the second paragraph after the general capture results (line 223-231). The remaining results have been slightly adjusted to keep fluency in light of the reordering of the key results.

17. Line 221: remove the 'd' (before Fig 4).

**Author response:** 'd' has been removed on line 255 before the reference to Fig 4.

18. TABLE 3: Include in the table legend what the model is of.

**Author response:** We appreciate this comment. To implement the suggestion, we have added '...p- values for the final model, describing the factors influencing larval burdens on bank voles. Non-significant predictors are retained in the model because of their involvement in an interaction or other predictor variable which is significant.'

19. Also, it may be my print out but, there seems an overlap of text in the final column.

**Author response:** Regarding the tables, all 3 tables have been reformatted according to the suggestions of Reviewer #3. Please notify us if the overlap of text is still occurring in your version, but hopefully all tables should display correctly now.

20. DISCUSSION: Reads a little sporadic overall, like a long chain of thought, but mentions some good points.

**Author response:** Thank you!

21. Line 254-55: I'd remove this sentence.

**Author response:** I am unsure which sentence this is referring to. Did you mean the sentence “However, these studies represented laboratory conditions and it is reasonable to assume that field conditions in our study (i.e. diurnal and nocturnal temperature fluctuations, as well as differences in humidity and light regime) could have affected the behavior of larvae and the duration of attachment after the death of a host..” on lines 254-257 of the original manuscript?

22. Page 11: Did your live trap prevent replete ticks from leaving the trap area? For example, Fig.1 indicates that the covered Ugglan / Sherman-type trap was used. Was the inside of traps examined for ticks which had dropped off? / Could the closed trap retain ticks (and humidity) better than the lethal trap.

**Author response:** The inside of the live traps (i.e. trap floor, and nesting material) was not examined for dropped off ticks. Neither trap type would prevent engorged ticks from leaving. In order to address this concern, we have added a sentence for clarification on lines 328-329. It is doubtful that humidity would have been retained more in a live traps compared to lethal trap. The roof covering would prevent precipitation from getting in, but the sides of the trap remain open, hence there would always be enough draft through the trap to cancel any difference in humidity.

23. Lines 284-287: This sentence seems to discuss two separate topics, and could be separated.

**Author response:** We agree. The sentence on lines 345-348 has been split into two. The first sentence now covers the lizards as cited in Pollock (2012), the second sentence refers to the findings in our study.

24. Mention grooming behavior e.g. of live captured mice, or grooming differences between sexes.

**Author response:** Thank you for bringing up this important point. It is true that one of the reasons that the burden in live traps could be smaller than in the lethal traps, is that aside from ticks reaching full engorgement, voles would have had additional time to potentially remove some of the burden through self-grooming. We have implemented your suggestion, by mentioning the previous point on lines 338-340.

25. Did you consider controlling for humidity in your traps (to avoid drop-off)?

**Author response:** No we did not. The reason for this is feasibility. In total we deployed 400 traps per study area (800 traps in total). Accounting for humidity inside the traps would require an equal amount of humidity loggers. Additionally, lethal traps are of open design, and live traps are semi-open. Humidity inside the traps is therefore assumed to be equal to the humidity outside.

26. How would tick life stage be expected to affect if there was a difference between lethal and live trap tick burdens across the seasons? You mention adults on deer, but what about nymphs?

**Author response:** I. ricinus nymphs generally do not parasitize small rodents but rather intermediate size animals. As with adult hosts (cervids), there is no data available on numbers of nymph hosts (e.g. foxes, hares etc.). We did not address this in the paragraph on lines 372-374. Furthermore, we feel there there is no reason to expect the non-significant differences in larval burdens between trap types to influence the nymphal population.

27. Lines 312-313: Suggest 'Performing a survey of cervids was outside the scope of this study,'

**Author response:** Thank you for the suggestion. We have implemented your suggestion by changing this to your recommendation on line 374-375.

28. Please discuss what your finding of a significant burden difference in study area implies? e.g. should different regions of the country be examined.

**Author response:** The effect of study area is now discussed further in the revised manuscript on lines 371-372 and 379-381.

29. Lines 319-320: I'm wondering how you can state this, when your methodology only describes one temperature recorder placed between the two grid types.

**Author response:** We are unsure of which sentence you are referring to in particular. However, we have 10 temperature loggers at every altitude and at every altitude station the live and capture plots are approximately 100m from eachother. The loggers being in the middle between the two (approximately 50m from each plot), we therefore assume the recorded temperatures are very similar to the actual temperatures inside the plots.

30. Lines 326-329: Check this long sentence. Line 329 - replace 'continued' with 'continuing'.

**Author response:** We agree. The sentence on lines 345-348 has been split, and the word 'continued' changed to 'continuing'.

31. In general, check places where commas should be inserted.

**Author response:** Thank you for the suggestion. Aside from the commas in the abstract, several additional commas have been added in the manuscript body.

32. Line 334: Still haven't read why killing the animals is necessary for this particular tick monitoring activity.

**Author response:** Thank you for this point of concern. As you mentioned earlier in your point 2., additional lines 143-148 have been added to address this concern.

33. FIGURES: I realize myself what the axes/legends are referring to, but suggest including making this obvious. For example, label '"tick" burden', rather than burden; label 'No. of "Vole" Captures'. Also describe more fully within legends what is being shown in the figure/table.

**Author response:Author response:** Clarity to the reader is of primordial importance. We therefore decided to implement your suggestion. Y axes on Fig 2, 3 and 4 have been adapted as per your recommendation. Additional information has also been added in each of the figure legends for clarification.

**Reviewer #2**

1. Authors aimed to test whether trapping method, lethal or live traps, used to capture bank voles (Myodes glareolus) had significant impacts on the tick burden size at time of collection. The study was conducted as a sub-study of a larger project over a period of 2 years in Norway and decidedly focused only on infestation burden of Ixodes ricinus larvae on the captured rodents. They found that while lethal traps did result in larger burden sizes, a significant difference was not obtained (p = 0.420) and suggest the use of lethal over live traps to avoid causing the animal stress during capture.

**Author response:** Thank you for the summary. This is precisely the core message of the study we aim to convey to the reader with this study.

2. While the study did mention that other species of tick were collected from captures bank voles, they choose to focus solely on I. ricinus larvae, though it is not clear why this decision was made nor was it stated what other species of tick were found on the captures bank voles.

**Author response:** This is a good point. The other species of ticks we encountered is the vole tick *Ixodes trianguliceps*. The reason why we did not include this species in this study is two-fold: firstly, the infestation rates of both *I. ricinus* and *I. triangulicpes* are the main focus of another study within this project. Secondly, *I. trianguliceps* is a nidiculous nest-dwelling specialist tick species. It does not quest in open vegetation and hence has a different ecology compared to *I. ricinus*. In order to remove potential confounding variables regarding *I. trianguliceps*, we chose to focus solely on the infestation rates of *I. ricinus*. Additionally, *I. ricinus* comprised the majority of all ticks collected from voles. In order to address your comment in the revised manuscript, we mentioned the observation of *I. trianguliceps* and briefly mentioned the reason for omitting this species in this study on lines 182-184.

3. Additionally, while environmental conditions were mentioned as serving as a defining factor in tick activity and therefore burden size, I am left wondering what was so different about 2018 that it impacted the burden size the way it did and it is my opinion that it would be to the benefit of the reader for the authors to include their judgment on why this occurred.

**Author response:** Indeed, 2018 had significantly fewer ticks than 2017. We suspect the reason for this is that 2018 was an exceptionally dry year in Norway. In order to address your concern in the revised manuscript, we have included humidity as a potential explanation in the discussion, adding the difference in precipitation days for both years in each study areas on lines 386-389.

4. It is my opinion that this study serves dual importance. While not only ethically important to reduce the suffering/stress of a captured animal, it is advantageous to strive for studying animals in the most natural conditions possible—natural conditions in this study being a live host for the tick to feed upon. Especially when feeding behavior of ticks on live versus dead hosts is not well studied, these findings provide some insight into what conditions may be factoring into their drop-off behavior. Overall, I believe that the study was well conducted, and that the paper is well written, yielding insights beneficial to future studies on tick burden size and small mammal capture.

**Author response:** Your comment is greatly appreciated. We aimed to determine which of the two capture methods would yield the highest precision, and if animal suffering could be reduced. Our hope is that this study might aid future researchers investigating tick burdens on rodents on which capture method is the most suitable to meet their needs.

**Reviewer #3**

SUMMARY: There are several methods that have been used to estimate tick burden (i.e., number of ticks per host), however comparative studies to evaluate methods are rare. This manuscript compares the efficacy of two forms of trapping methods to estimate larval Ixodes ricinus tick burdens on the bank vole, an important rodent reservoir species of several tick-borne diseases in Europe. In general, the manuscript was well written, the methods used to test the comparison and analyze the results were appropriate. The conclusions are generally sound. This paper will assist other researchers in choosing the most suitable sampling method for addressing their specific research objectives.
The following critique is intended to improve the manuscript.

**Author response:** We are grateful for your review. Our hope is to contribute to filling in the gap in comparative studies in capture methods in this field. We welcome reviewers' comments and critiques as an opportunity to improve on the quality of our manuscript.

MAJOR CRITICISM:

1. I do not know if this was the authors' fault, but Tables embedded within the text were formatted poorly, resulting in misaligned numbers or entire columns of data being truncated, making review of the manuscript unnecessarily difficult. For example: Table 2. Data for Year 2018, Summer and Autumn are not present; the columns lie outside the margin of the page. Likewise, Table 3, Column 5. There are typed-over numbers, some which completely obscure the p-values. What are these extra numbers and where do they belong? If they are extension of the p-values, please consider abbreviating p-values to 3 decimal places.

**Author response:** Thank you for this important remark. As reviewer #1 mentioned, table 3 had some readout problems, we do not know how this occurred. Regardless, we have reformatted all tables in the revised manuscript. Table 1 and Table 2 have been reconstructed vertically. Hopefully all data should now be easily readable. Please notify us if this would not be the case. Table 3 has also been reconstructed and reformatted, according to your remark 9. (see below) and the order of the covariates have been rearranged in order of significance. Also here, we encourage you to please notify us if there are still problems with readability in any of the tables.

2. Table 3 indicates a significant interaction effect between trap type and season.

Figure 2 indicates larval Ix. ricinus are most abundant in the spring, indicating that springtime is the most important season to sample voles for larval ticks in Norway.

Figure 2 also indicates that during the springtime, voles captured by lethal traps had higher mean burdens of larval ticks than voles captured alive. Indeed, standard error bars around the means did not overlap. Did authors test for statistical significance in mean tick burdens between trap types for springtime ONLY (i.e., perform a t-test on log-transformed counts or nonparametric Wilcoxon signed rank test on untransformed counts)? If not, this should be done. If so, please report the statistical outcome.

**Author response:** Thank you for the suggestion. In light of your comment, we performed a post-hoc Wilcoxon on the untransformed data for each separate season. We opted for a non-parametric test as log transformation (or any other transformation method) does not normalize de the data, and because the dataset contains a lot of 0 values, log-transformation introduces infinities in R. We have added the results of the post-hoc procedure in the Results chapter on lines 230-231, showing that no significant differences in burdens were observed between capture methods between seasons, with the exception of autumn.

3. Were there any differences between trap types and tick infestation PREVALENCE (i.e., % of voles parasitized with larval ticks)? A brief sentence or two to state the results should be included.

**Author response:** Indeed this was not addressed in the initial manuscript. We appreciate your suggestion and have implemented this as a result. The differences in infestation prevalence between capture methods is minimal. We have included the percentages of the infestation prevalence for each trap type on lines 220-221.

MINOR COMMENTS:

4. Authors would benefit from reading (and citing) the recent review article concerning methods for estimating tick burdens on host animals – see: Lydecker HW, et al. 2019. Counting ticks is complex: A review and comparison of methods. J. Med. Entomol. 56: 1527-1533.

**Author response:** Thank you for recommending this article. It proved very informative. Lydecker et al. gives a very good summary of surveying methods for ticks, as well as the benefits/disadvantages of each method. We have implemented this paper, and cited papers therein in the revised version of our manuscript in the Methods chapter (*Host capturing*) on lines 143-148.

5. Page 10, Table 1. Please add another column to this table at the far right-hand side to indicate the total number of voles for row 4 (Lifjell), row 5 (Laerdal), and row 6 (Totals). Heading for this new column should be “Total”.

**Author response:** An additional column has been added to Table 1 as per your suggestion indicating the Total captures for every row in the table.

6. Line 317 states “Burden size increased with temperature”. But Fig. 2 shows the highest burden sizes were in the Spring, not the Summer. Typically, ambient temperatures are LOWER in the Spring than in the Summer. Was this not the case in Norway during 2017 and 2018? Perhaps, authors should state somewhere in the paper, what were the average daily temperatures at each location during May 20th – 30th, July 20th – 30th, and September 20th – 30th. If necessary, re-assess the statement that “burden size increased with temperature”.

**Author response:** Thank you for your accurate point. The statement “burden size increases with temperature” is because the final model shows that temperature does indeed have a positive effect on burden size. The reason why this is not reflected in Fig.2, is because there are other influential predictors in the final model that have a stronger negative effect on burden (e.g. season). Humidity was not considered in this study for logistical and feasibility reasons, and higher summer temperatures might also have lead to drier conditions, therefore negatively affecting tick activity.

7. Line 26. Abstract. The species name of the tick is misspelled.

**Author response:** Thank you for pointing this out. the name *I. ricinus* has been corrected on line 31.

8. Line 210. I do not think that a p-value of 0.01 indicates “borderline significance”. Please re-assess.

**Author response:** You are correct in stating that a p- value of 0.01 is not borderline significant. In fact, we noticed that the significance level is in fact. 0.02. We have adjusted this on line 225 and removed the term 'borderline'.

9. Page 11, Table 3. Please list the parameters in some meaningful way. Suggest listing the parameters in order of their significance – i.e., p-values.

**Author response:** We appreciate the suggestion. Table 3 has been reordered in order of significance. Additionally, the predictor variable names in the table have been refined somewhat for additional clarity.

10. Line 203. Values in parenthesis – (4.8 and 1.2, respectively). Please indicate the units for these values – i.e., (4.7 and 1.2 ticks per vole, respectively).

**Author response:** Your suggestion has been implemented and 'ticks per vole' has been added as a unit on line 236.

11. Line 205-206. Something is missing at the end of this sentence. Suggest …”compared to 2017”.

**Author response:** We are afraid we do not know exactly which sentence you are referring to.

12. Lines 284 – 287. This sentence references Pollack (2012) study on lizards, followed immediately with reference to current study on voles. Recommend splitting the sentence into two sentences – one about lizards, the second about voles.

**Author response:** We agree. The sentence on lines 345-348 has been split into two. The first sentence now covers the lizards as cited in Pollock (2012), the second sentence refers to the findings in our study.

13. Line 230, Figure 4 caption. Should read … “Mean numbers of male and female voles captured per trap.”

**Author response:** Your suggestion has been implemented in the caption of Fig 4 (line 264), and changed to ' Number of male and female voles captured per trap type'. However, the numbers are absolutes, not means.

14. Line 234. Table 2 title. Please change the word “ticks” to “larval Ixodes ricinus ticks”.

**Author response:** Table 2 title has been changed to 'larval *Ixodes ricinus* ticks' instead of 'ticks' on line 286.
